# Genome-based assessment of antimicrobial resistance reveals the lineage specificity of resistance and resistance gene profiles in *Riemerella anatipestifer* from China

**DOI:** 10.1128/spectrum.03132-23

**Published:** 2024-01-03

**Authors:** Zhishuang Yang, Mingshu Wang, Renyong Jia, Shun Chen, Mafeng Liu, Xinxin Zhao, Qiao Yang, Ying Wu, Shaqiu Zhang, Juan Huang, Xumin Ou, Sai Mao, Qun Gao, Di Sun, Bin Tian, Yu He, Zhen Wu, Dekang Zhu, Anchun Cheng

**Affiliations:** 1 Research Center of Avian Diseases, College of Veterinary Medicine, Sichuan Agricultural University, Chengdu, Sichuan, China; 2 Key Laboratory of Animal Disease and Human Health of Sichuan Province, Chengdu, Sichuan, China; 3 International Joint Research Center for Animal Disease Prevention and Control of Sichuan Province, Chengdu, Sichuan, China; 4 Engineering Research Center of Southwest Animal Disease Prevention and Control Technology, Ministry of Education of the People’s Republic of China, Chengdu, Sichuan, China; 5 Key Laboratory of Agricultural Bioinformatics, Ministry of Education of the People’s Republic of China, Chengdu, Sichuan, China; China Agricultural University, Beijing, China

**Keywords:** *Riemerella anatipestifer*, antimicrobial resistance, antimicrobial resistance gene, whole-genome sequencing

## Abstract

*Riemerella anatipestifer* (*R. anatipestifer*) is an important pathogen that causes severe systemic infections in domestic ducks, resulting in substantial economic losses for China’s waterfowl industry. Controlling *R. anatipestifer* with antibiotics is extremely challenging due to its multidrug resistance. Notably, large-scale studies on antimicrobial resistance (AMR) and the corresponding genetic determinants in *R. anatipestifer* remain scarce. To solve this dilemma, more than 400 nonredundant *R. anatipestifer* isolates collected from 22 provinces in China between 1994 and 2021 were subjected to broth dilution antibiotic susceptibility assays, and their resistance-associated genetic determinants were characterized by whole-genome sequencing. While over 90% of the isolates was resistant to sulfamethoxazole, kanamycin, gentamicin, ofloxacin, norfloxacin, and trimethoprim, 88.48% of the isolates was resistant to the last-resort drug (tigecycline). Notably, *R. anatipestifer* resistance to oxacillin, norfloxacin, ofloxacin, and tetracycline was found to increase relatively over time. Genome-wide analysis revealed the alarmingly high prevalence of *bla*_OXA_-like (93.05%) and *tet*(X) (90.64%) genes and the uneven distribution of resistance genes among lineages. Overall, this study reveals a serious AMR situation regarding *R. anatipestifer* in China, with a high prevalence and high diversity of antimicrobial resistance genes, providing important data for the rational use of antibiotics in veterinary practice.

**IMPORTANCE:**

*Riemerella anatipestifer (R. anatipestifer*), an important waterfowl pathogen, has caused substantial economic losses worldwide, especially in China. Antimicrobial resistance (AMR) is a major challenge in controlling this pathogen. Although a few studies have reported antimicrobial resistance in *R. anatipestifer*, comprehensive data remain a gap. This study aims to address the lack of information on *R. anatipestifer* AMR and its genetic basis. By analyzing more than 400 isolates collected over two decades, this study reveals alarming levels of resistance to several antibiotics, including drugs of last resort. The study also revealed the lineage-specificity of resistance profiles and resistance gene profiles. Overall, this study provides new insights and updated data support for understanding AMR and its genetic determinants in *R. anatipestifer*.

## INTRODUCTION

China is one of the world’s largest producers and consumers of antibiotics, which are widely used to treat infections in humans and livestock. They are also used as prophylactic drugs and growth promoters in livestock. China reportedly accounted for 45% of the global use of veterinary antimicrobial agents in 2017. Despite a predicted reduction to 43% by 2030, China will remain the largest consumer of the stated drugs (1). Antimicrobial resistance (AMR) caused by the overuse and misuse of antibiotics has become a global concern, endangering the safety of livestock products and the health of consumers (2, 3).

According to the Food and Agriculture Organization of the United Nations (https://www.fao.org/faostat/en/#data/QCL) report, approximately 6.06 billion meat ducks were produced worldwide in 2021, with China accounting for approximately 68% of this total. Notably, the duck industry suffers significant yearly economic losses due to *Riemerella anatipestifer* (*R. anatipestifer*, RA), a Gram-negative bacterium that causes serious systemic infections and up to 75% mortality in a variety of poultry (e.g., duck, goose, and turkey flocks) globally (4–6). Despite *R. anatipestifer* vaccine development (7), antibiotics remain the primary measure for *R. anatipestifer* infection control, as cross-protection across heterologous serovars remains unavailable (8). However, the horizontal transfer of antimicrobial resistance genes (ARGs) (9) and severe resistance status (10–12) reduce antibiotic efficacy in treating *R. anatipestifer* infections. *R. anatipestifer* has been recognized as the natural reservoir or potential source of transmission for certain ARGs, such as *tet*X and various beta-lactamase genes (13–16). Furthermore, while several antibiotics are not standard treatment for *R. anatipestifer*, resistance genes or mutations have been reported in *R. anatipestifer*, including the oxacillin resistance gene *bla*_OXA_ and the *rpoB* gene mutation that causes rifampicin resistance (17, 18). Several studies have explored *R. anatipestifer* resistance (12, 19, 20), but their scale was small and only covered a few provinces in China. Meanwhile, conventional studies have assessed AMR based only on phenotype, lacking insights into the corresponding genetic determinants. Hence, a comprehensive understanding of the current AMR profile of *R. anatipestifer* in China is essential for the rational use of antibiotics in veterinary practice and for monitoring the potential transmission of ARGs.

In this study, the AMR of *R. anatipestifer* isolates collected from 22 provinces in China and other locations globally was assessed. Furthermore, whole-genome sequencing (WGS) of these isolates enabled an unprecedented scale of investigation into the potential relationship between resistance phenotypes and genetic determinants.

## MATERIALS AND METHODS

### Sample collection, isolation, and identification

Overall, *R. anatipestifer* field isolates were collected between 1994 and 2021 from 22 provinces in China. Poultry brain, liver, and tracheal swabs (resuspended in phosphate buffer saline solution) were collected; single colonies were then isolated by streaking onto tryptic soybean agar plates with 5% sheep blood. In this study, one representative *R. anatipestifer* isolate from each farm was selected based on location and time to establish the sample set for subsequent analysis. In addition, 15 *R*. *anatipestifer* strains were obtained from the American Type Culture Collections (ATCC) and Culture Collection University of Gothenburg. All *R. anatipestifer* isolates were confirmed by RA-specific and 16S rRNA PCR assays, as described previously (21, 22).

### Antimicrobial susceptibility testing

Following the Clinical and Laboratory Standards Institute (CLSI, USA) guidelines (CLSI M100, 31st edition, 2021), minimum inhibitory concentration (MIC) values were calculated using the broth microdilution method to assess the AMR of 417 *R*. *anatipestifer* isolates. Notably, 20 different antimicrobial agents in 10 categories (purchased from Meilunbio, Dalian, Liaoning, China) were included in the assay: aminoglycosides [gentamicin (GEN), kanamycin (KAN), and streptomycin (STR)], amphenicols [chloramphenicol (CHL) and florfenicol (FFC)], cephalosporins [cefalotin (CEF), cefotaxime (CTX), ceftazidime (CAZ), and cefepime (FEP)], cephamycins [cefoxitin (FOX)], macrolides [erythromycin (ERY) and azithromycin (AZM)], penicillins [ampicillin (AMP)], quinolones [norfloxacin (NOR) and ofloxacin (OFX)], rifampin (RIF), sulfonamides [trimethoprim (TMP) and trimethoprim/sulfamethoxazole (SXT)], and tetracyclines [tetracycline (TET) and tigecycline (TGC)]. *Escherichia coli* ATCC 25922 was used as a quality control strain in susceptibility testing. The experiments were repeated in triplicate. Results were interpreted using breakpoints of the European Committee on Antimicrobial Susceptibility Testing (version 12.0, 2022) and CLSI guidelines.

### Whole-genome sequencing

The TIANamp Bacterial Genomic DNA Kit (Tiangen Biotech Co., Ltd., Beijing, China) was used to extract the genomic DNA of individual bacterial isolates cultured overnight in tryptic soy broth medium at 37°C with shaking (180 rpm), and the DNA concentration was determined using the NanoDrop 2000 spectrophotometer (Thermo Fisher Scientific, Waltham, MA, USA). Genomic DNA of eligible quality was sent on dry ice to Beijing Genomics Institute (Shenzhen, China) and sequenced on the MGISEQ-2000 platform using a paired-end (PE 150 bp) library at an average coverage of 120 times per genome. Raw reads were filtered by Fastp (version 0.23.2) (23) with default parameter and assembled by SPAdes (version 3.15.0) (24) with the following parameters: --careful -k 21,33,55,77,99,111. The completeness and contamination of genomes were estimated using “checkm taxonomy_wf -genes” command in CheckM v1.1.10 (25).

### Bioinformatic analysis

Genome annotation and gene prediction were conducted using the NCBI Prokaryotic Genome Annotation Pipeline (version 2022-10-03.build6384) (26) and Prokka (version 1.14.6) (27). Genome clustering was performed using dRep (v2.0.0) (28) based on genomic distance (>2,000 variant sites, 99.9% similarity threshold). ARGs were identified using the NCBI AMRFinder plus (version 3.10.24) (29). Previously reported antimicrobial resistance mutations (ARMs) (18, 30) responsible for quinolones and rifampicin resistance were detected using snippy (version 4.6.0, https://github.com/tseemann/snippy), which is based on BWA-mem (31) and freebayes (32) to call single nucleotide polymorphisms (SNPs). Multi-locus sequence type (ST) of the genomes was identified using mlst v2.19.0 (https://github.com/tseemann/mlst) against the PubMLST database (https://pubmlst.org/) (33). The pan-genome inference was performed using Roary v3.13.0 (34) with a 95% sequence identity threshold. A core-genome-based neighbor-joining (NJ) tree was conducted using PopPUNK v2.4.0 (35). The phylogenetic lineages were inferred from the core genome alignment using Fastbaps v1.0.8 with the optimized BAPS prior algorithms (36).

### Statistical analyses

All statistical analyses were performed in R version 4.2.0 (R project for statistical computing, http://www.r-project.org/). The level of agreement between the AMR genotype and phenotype was assessed using Cohen’s kappa test (37), and the concordant results indicated that the isolate phenotype and genotype were both resistant and susceptible. The Kruskal-Wallis rank-sum test was applied for multiple group comparisons and the Wilcoxon rank-sum test was applied for the comparison between groups. Differences in the distribution of resistance genes between lineages were compared using the chi-square test or Fisher’s exact test, as appropriate.

## RESULTS

### Characterization of the examined *R. anatipestifer*

**Fig 1:**
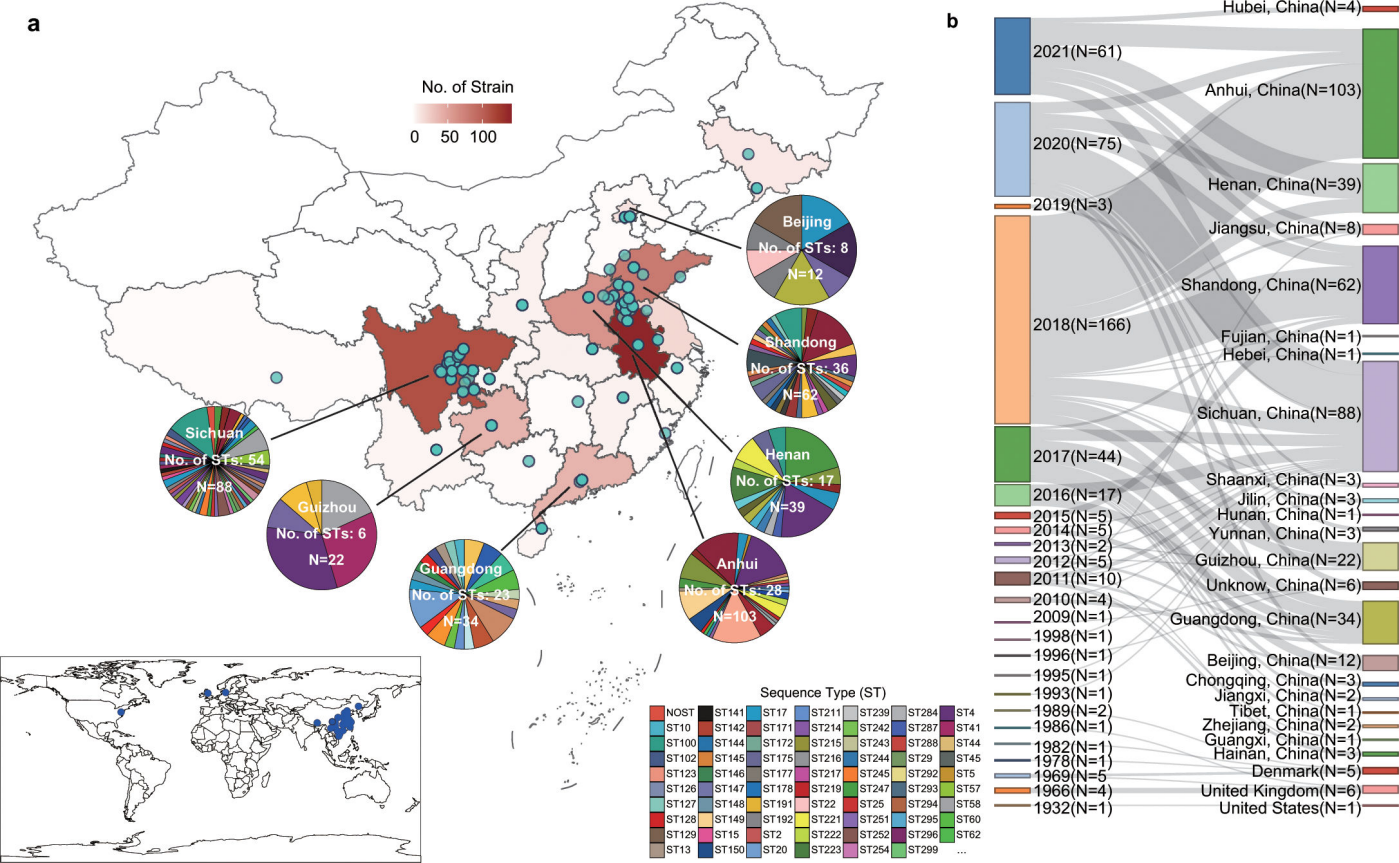
Geographical and temporal information of tested *Riemerella anatipestifer* isolates. (a) Geographical information of tested samples. The blue dots indicate individual samples. The pie charts represent the multilocus ST composition of the major source provinces (the number of isolates exceeds 10). [The open-source packages leaflet (version 2.1.2, https://leafletjs.com) and leafletCN (version 0.2.1, https://github.com/Lchiffon/leafletCN) within R software (version 4.3.1, https://www.r-project.org/) were used to visualize the geographic information.]. (b) Sankey diagram showing the temporal and geographical distribution of isolates.

The isolates examined in this study were collected mainly across the Anhui, Sichuan, Shandong, Henan, and Guangzhou provinces in China (Fig. 1a; Table S1). In China, the earliest known isolate was RA-CH-1 isolated in Sichuan province in 1993 (Fig. 1b). A total of 415 strains (99.52%) could be categorized into 153 STs based on multi-locus sequence typing (MLST) analysis results, with ST4 (37/415), ST91 (31/415), ST100 (19/415), ST160 (16/415), ST99 (14/415), ST58 (12/415), ST98 (11/415), and ST149 (10/415) being the most prevalent STs. Sichuan (number of STs = 54), Shandong (number of STs = 36), Anhui (number of STs = 28), Guangdong (number of STs = 23), and Henan (number of STs = 17) provinces were the top five in terms of ST diversity (Fig. 1a; Table S1).

### Antimicrobial resistance phenotype

**Fig 2:**
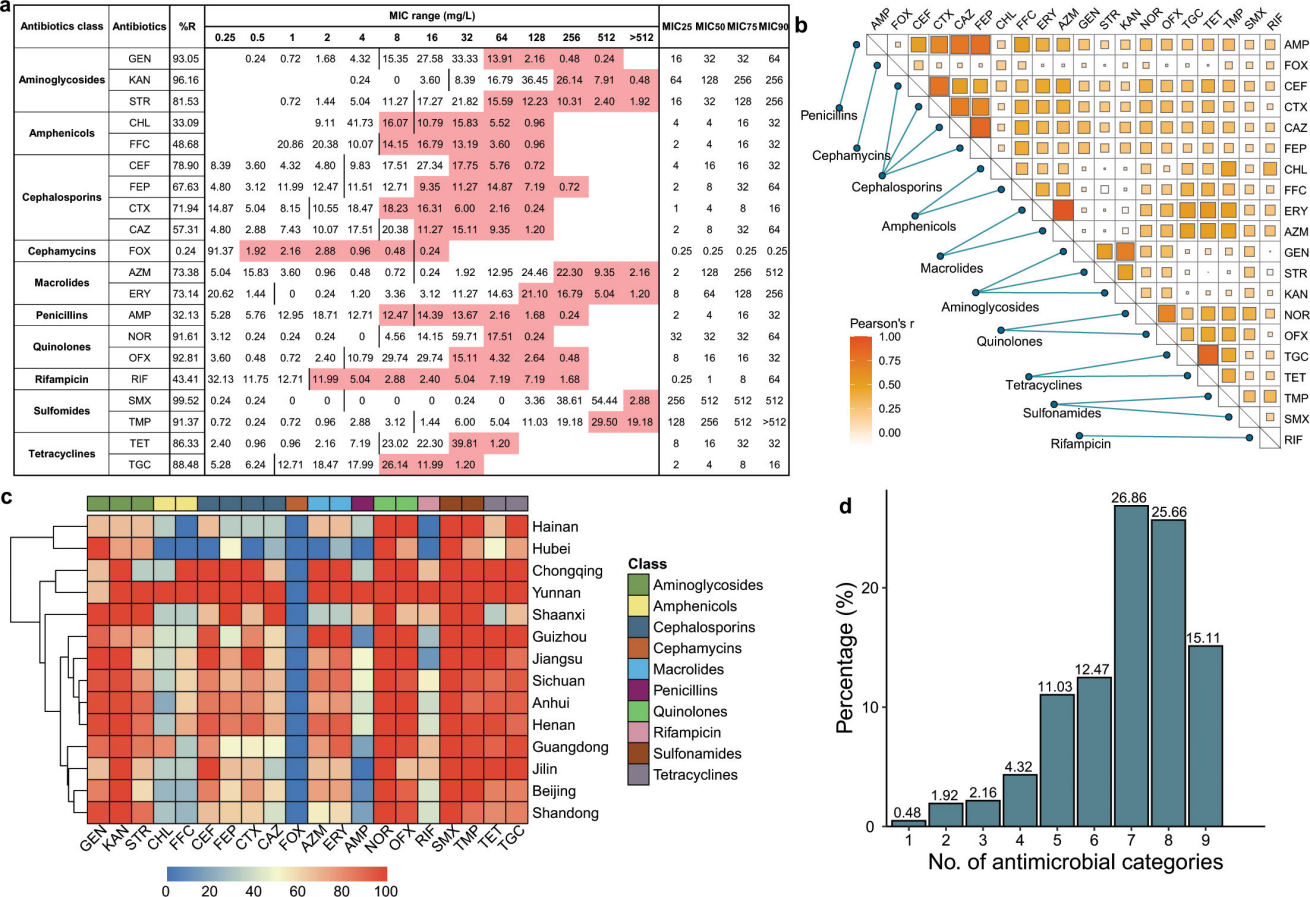
Antimicrobial resistance characteristics of examined *R. anatipestifer* isolates. (a) Distribution of MIC values among the examined *R. anatipestifer* isolates (*n* = 417) against different antimicrobials. The tested antimicrobials in the second column are as follows: GEN, KAN, STR, CHL, FFC, CEF, CTX, CAZ, FEP, FOX, ERY, AZM, oxacillin (OXA), AMP, NOR, OFX, RIF, TMP, SMX, TET, and TGC. The vertical bar indicates the MIC breakpoints for each antimicrobial, the left cell (less than or equal to) is regarded as susceptible, and the right cell (greater than) is regarded as resistant (intermediate values are designated as resistant). The pink area indicates a value exceeding MIC50. (b) The correlation of the MIC values of different antibiotics. The size and color of the square represent the Pearson correlation coefficient (R). (c) AMR (percentages) among different provinces; the color of the cell corresponds to the percentage prevalence of each province. Note: only provinces with three or more isolates are shown in the heatmap. (d) Distribution of multidrug-resistant isolates.

Antimicrobial susceptibility testing (AST) results showed that nearly all strains (99.52%, 415/417) were resistant to sulfamethoxazole (SMX), with MIC_25_ and MIC_50_ up to 256 and 512 mg/L, respectively, followed by kanamycin (96.16%), gentamicin (93.05%), ofloxacin (92.81%), norfloxacin (91.61%), and trimethoprim (91.37%), whereas the lowest levels of resistance were observed in cefoxitin (0.24%) (Fig. 2a). Most isolates were resistant to tetracycline (86.33%, 360/417) and tigecycline (88.48%, 369/417), and a considerable number of isolates were highly resistant to tigecycline (MIC ≥ 4 mg/L; 64.77%, 239/417). The remaining antibiotic resistance rate is between 32.13% and 81.53% (Fig. 2a). Correlation analysis revealed that the MIC values of different antibiotics were correlated (Pearson correlation coefficient > 0.3) (Fig. 2b), and further analysis of the resistance profiles revealed 49 resistance patterns in the studied isolates, of which 95.45% (398/417) were resistant to four or more antimicrobial classes [multiple-drug resistance (MDR)] (Fig. 2d). Resistance rates to aminoglycosides, macrolides, quinolones, sulfonamides, and tetracyclines were quite high in most regions but relatively low for amphenicols, cephalosporins, penicillins, and rifampins, in particular, cefoxitin-resistant strains were detected only in Guizhou (Fig. 2c). Notably, more than half of the strains tested (67.63%, 282/417) were resistant to seven or more antimicrobial classes (Fig. 2d), and the predominant multidrug-resistant phenotypic pattern was “Aminoglycosides-Amphenicols-Cephalosporins-Macrolides-Quinolones-Sulfonamides-Tetracyclines” (15.83%, 66/417) (Table S1).

**Fig 3:**
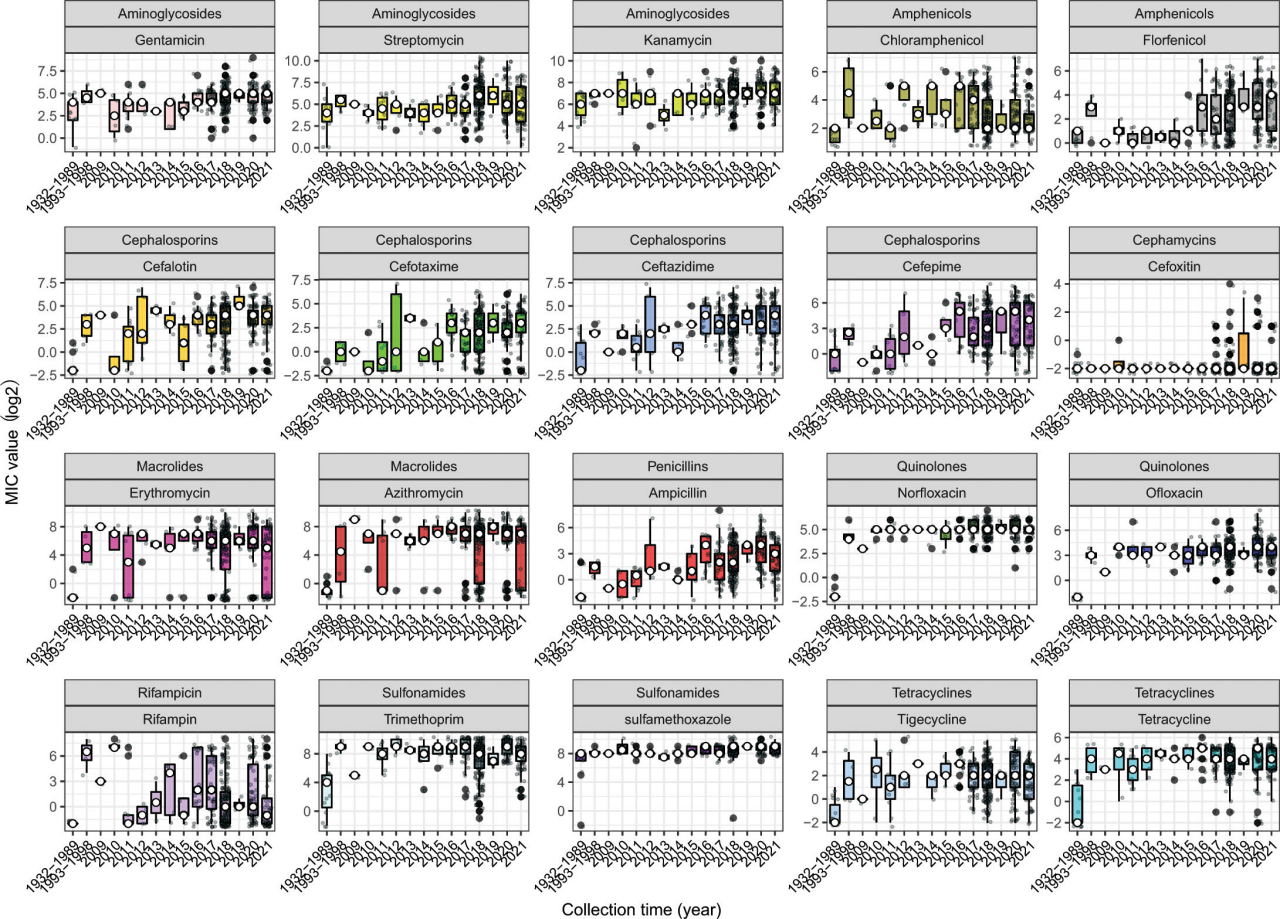
Distribution of MIC values of isolates at different collection times. Early strains were combined due to their scarcity. MICs are shown on a log2 scale.

We also compared the antimicrobial susceptibility of strains isolated at different times. The early isolates (prior to 1990) were relatively sensitive to all antibiotics except for sulfamethoxazole (Fig. 3). Furthermore, the results indicate that strains isolated between 1993 and 1998 already showed a high level of antibiotic resistance, except for cefoxitin, which exhibited a relatively high level of resistance in recent years. Overall, the early isolates exhibited lower levels of resistance than more recent isolates (since 1993).

**Fig 4:**
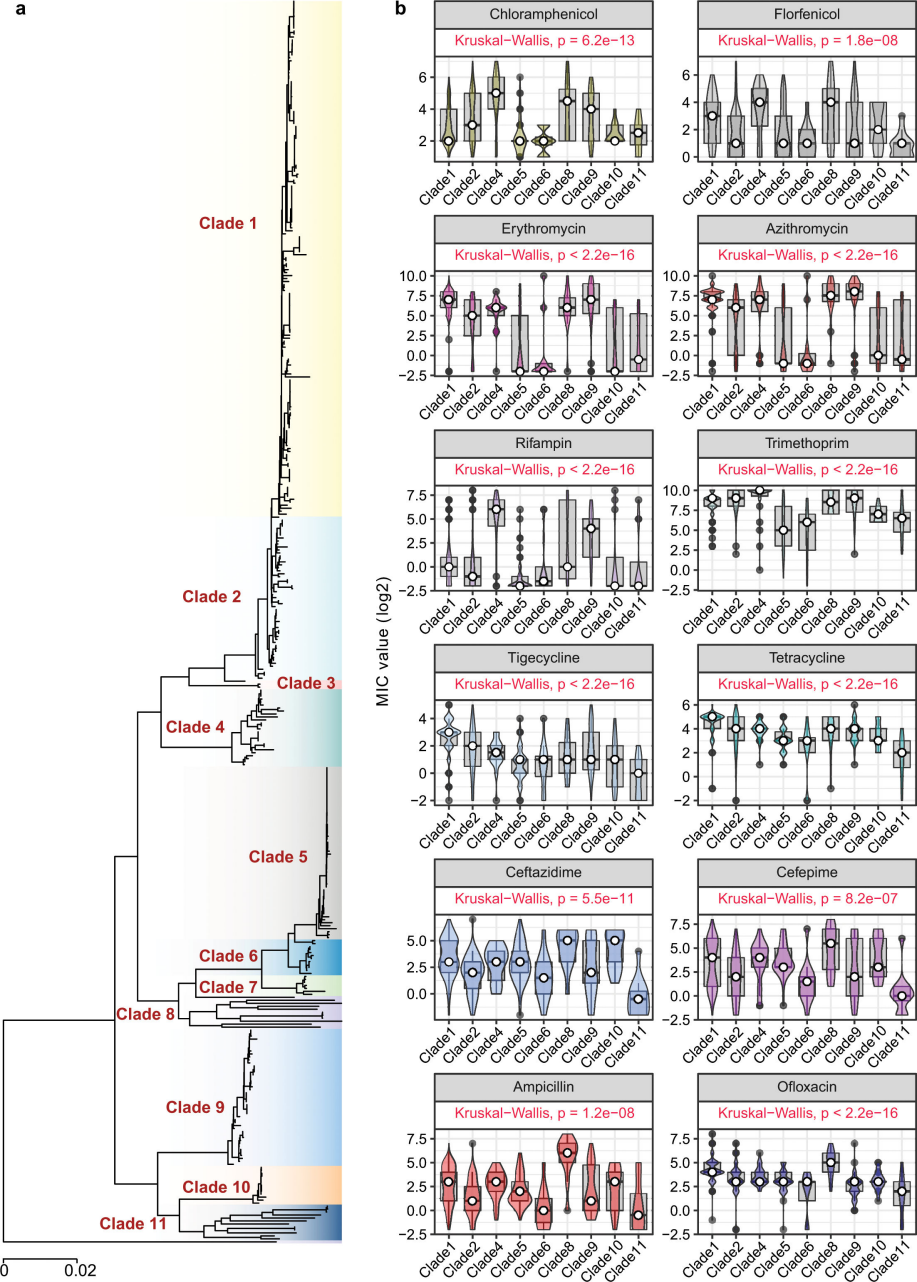
Comparison of MIC distributions among different lineages. (a) Phylogenetic analysis of all *R. anatipestifer* isolates. The core-genome-based NJ tree was conducted using PopPUNK. Eleven clades were identified according to the hierarchical Bayesian analysis by FastBaps (https://github.com/gtonkinhill/fastbaps) based on core genome alignment. (b) Comparison of MIC distributions among different lineages.

To investigate the relationship between phylogeny and antibiotic susceptibility, we compared MIC values across different lineage groups. Based on the core genome distance, all strains can be divided into three main branches with 11 lineage clades (Fig. 4; Table S1). Our findings revealed differences in susceptibility levels among strains belonging to different clades (focusing on groups with more than 10 observed samples). It is noteworthy that strains in clade 1 (41.49%, 173/417) exhibited relatively high MICs for several antibiotics, except for chloramphenicol and rifampicin, while strains in the adjacent clade 2 showed slightly lower MICs compared to clade 1 (Fig. 4; Fig. S1). Conversely, strains in clade 4 have higher MICs for amphenicols antibiotics and rifampicin. In addition, strains in clades 5 and 6 have relatively low MICs for several antibiotics, especially amphenicols, macrolides, rifampin, and trimethoprim, indicating a lower level of resistance. Interestingly, the strains in clade 11 (2.88%, 12/417) have lower MIC values for almost all antibiotics in comparison to other lineages (Fig. S1). The strains in clade 11 were mainly isolated from the respiratory tract (83.33%, 10/12), including one strain isolated from the respiratory tract of a black swan. Furthermore, we observed a decreasing trend in the resistance levels to tetracycline antibiotics and ofloxacin from clade 1 to clade 11 (Fig. 4).

### Genetic determinants associated with antimicrobial resistance phenotypes

**Fig 5:**
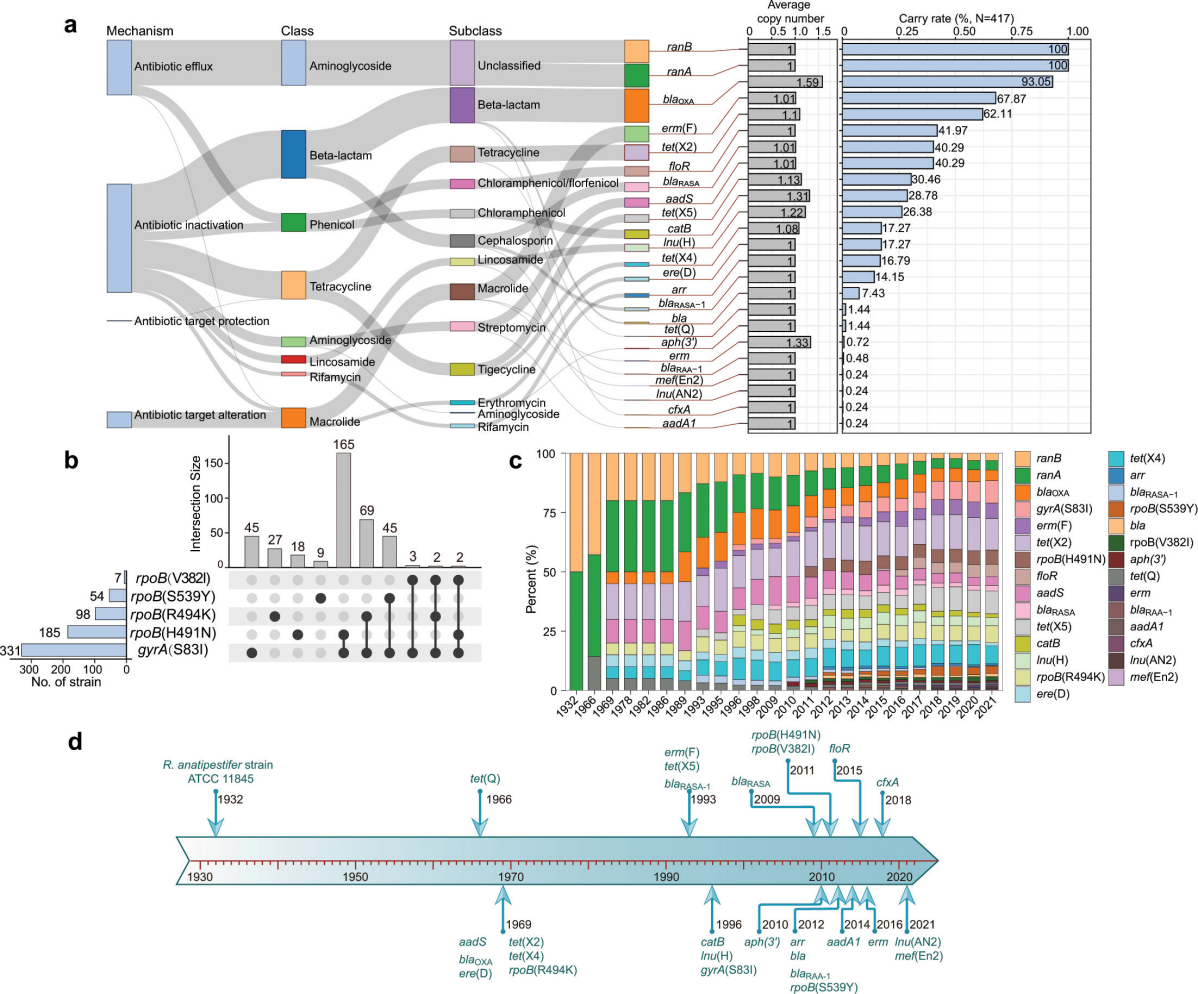
Genome-based identification of ARGs and ARMs in *R. anatipestifer*. (a) The category (left), average copy number, and carrying rate (right) of ARGs. (b) Statistics of ARMs previously reported in *R. anatipestifer*. (c) Cumulative proportion of ARGs and ARMs over time. (d) Timeline of ARGs and known ARMs in *R. anatipestifer*.

In this study, we used genomes with assembly completeness > 90% (upper quartile: 99.03%, median: 98.90%, and lower quartile: 95.28%) and contamination < 5% (upper quartile: 1.30%, median: 0.18%, and lower quartile: 0.1%; Table S1). Using these high-quality genomes, we performed genome-wide detection of ARGs. Genome-wide ARG results showed that all isolates were positive for the *ranA* and *ranB* genes (Fig. 5a; Fig. S2), which together encode an ABC-type efflux pump that confers resistance to aminoglycoside antibiotics. Notably, at least one of the beta-lactamase genes (*bla*_RASA_, *bla*_RASA-1_, *bla*_RAA-1_, *bla*_OXA_-like, *bla*, and *cfxA*) was detected in 96.40% (402/417) of the examined strains. The *bla*_OXA_-like gene had the highest carriage rate (93.05%, 388/417). A total of 36 isolates harbored three copies and 155 isolates harbored two copies, suggesting that multiple copy events of the *bla*_OXA_-like gene are prevalent in *R. anatipestifer*. Most tested strains harbored tetracycline resistance genes, with *tet*(X2), *tet*(X5), *tet*(X4), and *tet*(Q) present in 62.11%, 30.46%, 17.27%, and 1.44% of them, respectively. In addition, the identification of resistance mutations revealed that the *gyrA* (S83I) mutation, which confers high quinolone resistance, was detected in 79.37% of the tested strains (Fig. 5b). The *rpoB* (H491N) mutation, which confers resistance to rifampicin, was found in 44.36% of the tested strains. Importantly, 39.57% of the strains contained both *gyrA* (S83I) and *rpoB* (H491N) (Fig. 5b).

### Tracing the emergence of ARGs/ARMs in *R. anatipestifer* population

ARG/ARM-positive strains were tracked to explore the evolutionary patterns of ARGs/ARMs in the *R. anatipestifer* population. Our results indicated that the *tet*(Q) gene was already present in *R. anatipestifer* as early as 1966, and the *bla*_OXA_-like, *aadS*, *ere*(D), *tet*(X2), and *tet*(X4) genes were detected in strains isolated in 1969 (Fig. 5c and d). In China, positive strains of *bla*_RASA_, *erm*(F), and *tet*(X5) existed in 1993. The *catB* and *lnu*(H) genes were detected in strains isolated in 1996. The aminoglycoside resistance genes *aph*(3′) and *aadA1* were identified in strains isolated in 2010 and 2014, respectively. The *arr* gene, which confers resistance to rifampicin, first emerged in 2012. The earliest isolation of the *floR*-positive strain in this study occurred in 2015. Notably, a strain isolated in 2018 carries the *cfxA* gene, which encodes a protein capable of degrading cephalosporins and penicillins (38). In this study, the earliest *rpoB* (R494K) mutant strain was identified in 1969, and the earliest *gyrA* (S83I) mutant strain was identified in 1996 (Fig. 5d). Strains harboring the *rpoB*(H491N) and *rpoB*(V382I) mutations were first observed in 2011. Strains carrying the *rpoB*(S539Y) mutation were first isolated in 2012.

### The relationship between ARGs/ARMs and antimicrobial resistance levels in *R. anatipestifer*

**Fig 6:**
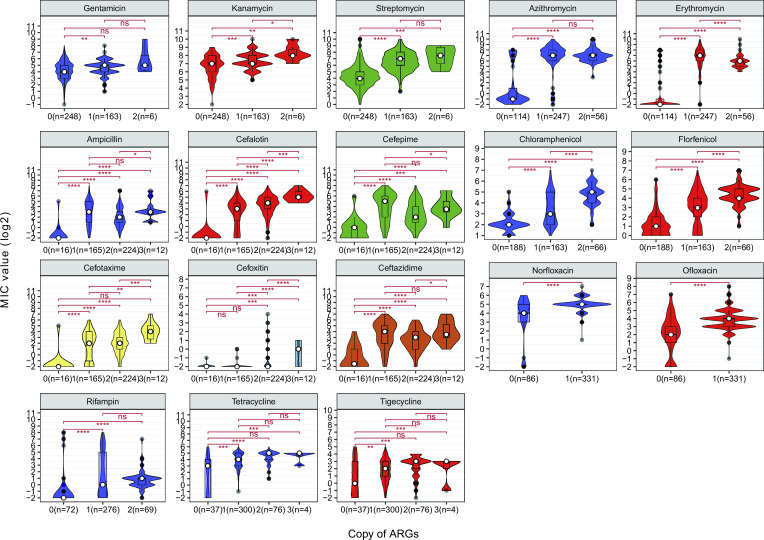
The impact of the copy number of acquired resistance genes on antibiotic MIC levels. Acquired resistance genes were identified using AMRFinder, and resistance genes of the same category were combined for statistical analysis. The Wilcoxon test was applied for the comparison between groups (ns, *P* > 0.05; *P ≤ 0.05; **P ≤ 0.01; ***P ≤ 0.001; and ****P ≤ 0.0001).

The assessment of MIC distribution differences between strains with and without ARGs revealed that strains with corresponding ARGs had significantly higher antibiotic MICs than ARG-negative strains (Wilcoxon rank-sum test, *P*-value < 0.05), except for cefoxitin (Fig. 6). We also analyzed the effect of the copy number of acquired resistance genes on the resistance profile of *R. anatipestifer*. Our findings indicated that MIC values for erythromycin, chloramphenicol, florfenicol, and cefalotin increased with an increase in the corresponding ARG copy numbers (Fig. 6). However, a higher number of ARG copies does not always indicate higher MIC values. For beta-lactam antibiotics, such as ampicillin, cefepime, ceftazidime, and cefotaxime, strains with a single copy of ARG generally have higher overall MIC values than strains with two copies, and strains with three copies of ARGs have even higher MICs than those with two copies. Interestingly, for tetracycline resistance, which is mainly mediated by the *tet*(X) gene, strains with two copies generally have higher MIC values compared to single-copy strains. However, strains with three copies do not seem to show a significant increase in resistance capability. This could be attributed to the limited number of samples with three copies.

Overall, a high concordance rate was observed between genotype and phenotype (Table 1). In addition, the concordance rate between the presence of at least one aminoglycoside ARG [*aadA1*, *aadS*, *aph*(3′), *ranA*, and *ranB*] and their corresponding phenotypes (gentamicin, streptomycin, and kanamycin) exceeded 81.53%. Considerable agreement (kappa index = 0.48–0.78) was found between phenotypic antimicrobial resistance and the presence of at least one ARG encoding resistance to macrolides and amphenicols. Specifically, the *floR* gene showed high concordance with the florfenicol resistance phenotype (kappa index = 0.82 and coincidence rate = 90.89%), followed by *erm*(F) against azithromycin (kappa index = 0.8). Moreover, moderate agreement (kappa index = 0.4 and 0.54, respectively) was observed between the presence of two ARMs and their corresponding phenotypes, which were *rpoB* (Arg494Lys) against rifampin and *gyrA* (Ser83Ile) against ofloxacin (Table S2).

**TABLE 1:** Concordance analysis of antimicrobial resistant genotype and phenotype^*a*^^,*g*^

ARG/ARM class	Antibiotic	True resistance^*b*^	Error resistance^*c*^	Error sensitive^*d*^	True sensitive^*e*^	Kappa index	*P* value	Level of agreement	Coincidence rate (%)^*f*^
Aminoglycosides	Gentamicin	388	29	0	0	–	–	–	93.05
Aminoglycosides	Streptomycin	340	77	0	0	–	–	–	81.53
Aminoglycosides	Kanamycin	401	16	0	0	–	–	–	96.16
Macrolides	Erythromycin	297	6	28	86	0.78	0	Substantial	91.85
Macrolides	Azithromycin	281	22	25	89	0.71	0	Substantial	88.73
Phenicols	Florfenicol	180	49	23	165	0.66	0	Substantial	82.73
Phenicols	Chloramphenicol	127	102	11	177	0.48	0	Moderate	72.9
Quinolones	Ofloxacin	328	3	59	27	0.4	0	Moderate	85.13
Tetracyclines	Tigecycline	346	34	23	14	0.25	0.01	Fair	86.33
Quinolones	Norfloxacin	330	1	71	15	0.25	0	Fair	82.73
Beta-lactams	Cefalotin	328	73	1	15	0.24	0	Fair	82.25
Tetracyclines	Tetracycline	337	43	23	14	0.21	0.01	Fair	84.17
Rifamycins	Rifampin	173	172	8	64	0.21	0	Fair	56.83
Beta-lactams	Cefotaxime	299	102	1	15	0.17	0.01	Slight	75.3
Beta-lactams	Cefepime	280	121	2	14	0.13	0.03	Slight	70.5
Beta-lactams	Ceftazidime	238	163	1	15	0.09	0.05	Slight	60.67
Beta-lactams	Oxacillin	139	262	1	15	0.03	0.18	Slight	36.93
Beta-lactams	Ampicillin	133	268	1	15	0.03	0.2	Slight	35.49
Beta-lactams	Cefoxitin	1	400	0	16	0	0.49	No	4.08

^*a*^ “–” implies that data are unsuitable for Kappa test.

^*b*^ True resistance: antimicrobial resistant phenotype resistant with corresponding AGRs/ARMs.

^*c*^ Error resistance: antimicrobial resistant phenotype susceptible but with corresponding AGRs/ARMs.

^*d*^ Error sensitive: antimicrobial resistant phenotype resistant but without corresponding AGRs/ARMs.

^*e*^ True sensitive: antimicrobial resistant phenotype susceptible and without corresponding AGRs/ARMs.

^*f*^ Coincidence rate: the ratio of the true resistance and true sensitive to the total number of strains.

^*g*^ No agreement (kappa index ≤ 0), none or slight (kappa index = 0.00–0.20), fair agreement (kappa index = 0.21–0.40), moderate agreement (kappa index = 0.41–0.60), substantial agreement (kappa index = 0.61–0.80), and almost perfect or perfect agreement (kappa index = 0.81–1).

### Differences in the distribution of ARGs across phylogenetic lineages

**Fig 7:**
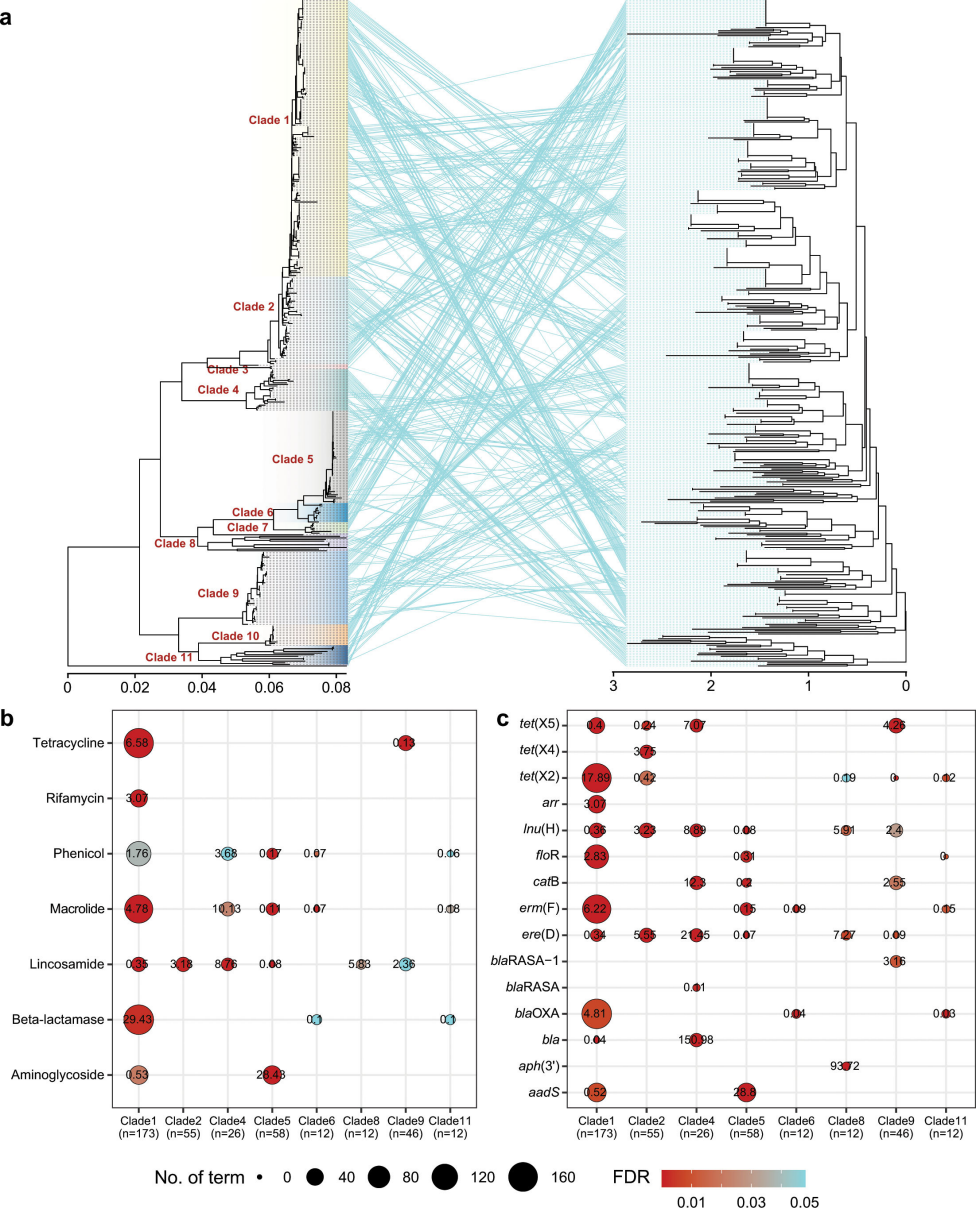
Comparison of differences in the distribution of resistance genes among lineages. (a) The tanglegram between core-genome-based NJ tree and NJ tree based on the distance of resistance gene profiles. (b) Differentially distributed ARGs among clades. (c) Differentially distributed ARG categories among clades. The numbers on the dots represent the odds ratio (OR) calculated using either the chi-squared test or Fisher’s exact test. OR > 1 indicates a positive correlation between the resistance gene and the phylogenetic clade, while OR < 1 indicates a negative correlation between the resistance gene and the phylogenetic clade.

To investigate the differences in the distribution of ARGs across different evolutionary lineages, we constructed an NJ distance tree based on the resistance gene profiles. We compared this tree with the NJ phylogenetic tree constructed using the core genome data. The results revealed that although the profiles of ARGs did not fully correspond to the phylogenetic structure of the strains, they still exhibited similar clustering effects in certain lineages, namely clade 4, clade 5, clade 6, and a portion of clade 1 (Fig. 7a; Table S3). This suggests a potential association between the ARG profiles of these strains and their phylogenetic background. To further confirm this correlation, we compared the distribution of ARGs within clades relative to the entire population. Interestingly, clade 1 is enriched (odds ratio > 1) with the highest diversity of antibiotic resistance genes, including beta-lactamase gene *bla*_OXA_-like, macrolide resistance gene *erm*(F), amphenicol resistance gene *floR*, rifamycin resistance gene *arr*, and tetracycline resistance gene *tet*(X2). However, aminoglycoside resistance gene *aadS* and lincosamide resistance gene *lnu*(H) are relatively depleted (odds ratio < 1) within clade 1 (Fig. 7b and c; Table S4). The findings are consistent with the higher MIC values observed in clade 1 strains for macrolides, tetracyclines, florfenicol, and beta-lactam antibiotics while exhibiting a relatively lower resistance phenotype for aminoglycosides (Fig. S1). Clade 2 is enriched with the *ere*(D) and *tet*(X4) genes, which may explain the high MIC values observed for macrolides and tetracyclines in this clade. Clade 4 is enriched with the *ere*(D), *tet*(X5), and *catB* genes (Table S4), which correspond well to the MIC phenotype, especially the *bla* gene, which could confer resistance to cefoxitin in clade 4 strains (Fig. S1). Clade 5 lacks resistance genes for macrolides, lincosamides, and phenicols, consistent with its susceptibility phenotype. Nevertheless, it is noteworthy that clade 5 exhibits an enrichment of the *aadS* gene (aminoglycoside resistance), which could explain the higher MIC values observed for gentamicin, streptomycin, and kanamycin in strains belonging to clade 5 (Fig. 7b and c; Fig. S1). Clade 6 is devoid of resistance genes to macrolides and beta-lactam antibiotics, thus strains in clade 6 are relatively sensitive to these two types of antibiotics. Although clade 8 is enriched only with the *aph*(3′), *ere*(D), and *lnu*(H) genes, the strains within this clade exhibit relatively high resistance to several antibiotics. Interestingly, the strains in clade 11 are devoid of resistance genes for macrolides, phenicols, and beta-lactam antibiotics. As expected, the isolates in this clade are more susceptible to almost all antibiotics.

## DISCUSSION

*R. anatipestifer* is an important pathogen of poultry and wild birds, and the presence of antibiotic resistance genes in *R. anatipestifer* has garnered increasing attention (13, 39, 40). However, a thorough comprehension of the potential resistance mechanisms in *R. anatipestifer* is currently lacking. Although similar studies have been undertaken, they have been limited to certain regions and periods (11). Therefore, the current study presents a geographically and temporally detailed assessment of AMR in *R. anatipestifer*. To comprehensively investigate AMR in *R. anatipestifer*, we conducted an analysis using WGS and phenotypic assays on a sample set consisting of 417 isolates. The isolates were collected from different regions of the world, with a focus on China, over a period of 89 years (1932–2021). This extensive collection allows for a comprehensive study of AMR trends over time and across various geographical regions. The AST results indicated an alarming AMR situation for *R. anatipestifer*, as corroborated by the prevalence of multidrug-resistant strains. Several recent regional studies have also shown a significant prevalence of multidrug-resistance strains in *R. anatipestifer* (11, 12, 14, 20). An earlier study showed that over 50% of 224 *R*. *anatipestifer* isolates collected between 1998 and 2005 was multi-resistant to up to 10 antimicrobials (10), and that 87.8% was resistant to trimethoprim/sulfamethoxazole. Gyuris et al. (41) revealed that over 70% of 185 *R*. *anatipestifer* isolates was resistant to flumequine, tetracycline, erythromycin, and streptomycin (41). A recent survey of 51 *R*. *anatipestifer* isolates in Shandong, China indicated the MDR of all tested isolates (12). These studies all indicate that multidrug-resistant *R. anatipestifer* is highly prevalent in different regions. Shockingly, the resistance rates for tetracycline and tigecycline were 86.33% and 88.49%, respectively (14). Using the same breakpoint, the tigecycline resistance rate in our study decreased to 39.33%. Notably, florfenicol (33.08%) and chloramphenicol (48.68%) showed relatively low resistance rates in this study, probably because chloramphenicol has been banned from animal-producing facilities in China since 2002 (42). In the present study, the linear regression analysis of collection time and MIC revealed that, as expected, the isolate susceptibility to the antimicrobials decreased with time, corroborating previous findings that selection pressure from antimicrobials could facilitate the expansion of drug-resistant bacteria (2, 43, 44).

WGS analysis, which provided in-depth data to understand the AMR in *R. anatipestifer*, revealed that the aminoglycoside resistance genes *ranA* and *ranB* were detected in all isolates, corroborating the findings of previous studies (12). In addition, 90.64% of the isolates harbored at least one of *tet*(X2), *tet*(X4), and *tet*(X5), which was consistent with previous PCR-based results (80.2%–100%) (12, 14, 45, 46). Our findings, in contrast to those of other studies (12, 14), suggested a high detection rate (93.06%) of the *bla*_OXA_-like gene, probably due to the excellent performance of the ARG detection strategy we employed (AMRFinderPlus was employed in combination with high-quality WGS data). Indeed, several of our previous studies have also shown a wide distribution of beta-lactamase resistance genes in RA (15, 16). Furthermore, recent studies have highlighted that *R. anatipestifer* can acquire resistance to beta-lactam antibiotics by acquiring plasmid pRCAD0416RA1 (17) and p20190305E2-2 (14) carrying the *bla* gene. Overall, the detection rate of ARGs based on WGS in our study was comparable to the early results based on PCR (12, 45). In this regard, the cost-effectiveness of WGS makes it an excellent method for ARG surveillance and study (47, 48).

The clinical use of penicillin in 1941 marked the beginning of the antibiotic era (49). The first aminoglycoside antibiotic, streptomycin, was introduced in 1948 (50). Perhaps, the mechanisms of antibiotic resistance have existed prior to the clinical use of antibiotics. In the present study, *ranA* and *ranB* were found to be inherent genes of *R. anatipestifer*, encoding an ABC-type multidrug efflux transporter that contributes to aminoglycoside resistance (51). The earliest *ranA-ranB*-positive known strain is the type strain ATCC 11845 isolated in 1932. Antimicrobials such as tetracycline and erythromycin were introduced into the clinic in the 1950s (52, 53), whereas *aadS, bla*_OXA_, *ere*(D), *tet*(Q), and *tet*(X) were present in *R. anatipestifer* as early as the 1960s. The rapid accumulation of ARGs in *R. anatipestifer* may benefit from its natural transformation capacity and horizontal gene transfer events (9, 54). Notably, there are few reports regarding plasmid-associated resistance genes in *R. anatipestifer* (14, 17, 55), and previous studies have indicated that resistance genes in *R. anatipestifer* are primarily located within chromosomal resistance gene clusters (14, 16, 39, 56). However, these resistance gene clusters have not been found to contain associated mobile genetic elements (14, 39, 56). The natural transformation capacity of *R. anatipestifer* may contribute to the formation of resistance gene clusters, but the specific mechanisms require further study.

A relatively high coherence (coincidence rate > 80%) existed between phenotypic and genotypic resistance in most antimicrobials, except for beta-lactam antimicrobials. However, the agreement test between genotype and corresponding phenotype was not excellent (kappa index = 0–0.78), except for the *flo*R gene encoding resistance to florfenicol and the *erm*(F) gene encoding resistance to azithromycin, corroborating previous report (12). Nevertheless, our results have shown that isolates carrying resistance genes have higher MIC values than those without resistance genes. This suggests that the detection of acquired resistance genes could reflect the antibiotic susceptibility of strains. In addition, this study has also revealed the frequent occurrence of multiple copy events of resistance genes in *R. anatipestifer*. However, it was observed that these multiple copy events do not always enhance the host’s resistance capacity. This could be due to the fact that resistance conferred by the single resistance gene is relatively low. This could also explain why the presence or absence status of a single resistance gene shows a lower consistency with the observed phenotypes. Therefore, the difference between phenotypic and genotypic resistance profiles implies that phenotypic assays remain the gold standard for evaluating the antimicrobial susceptibility of *R. anatipestifer*.

Horizontal transfer of ARGs is generally considered to be an adaptive mechanism of bacteria to specific environmental conditions, which is unlikely to be synchronized with the genetic background of the core genome. However, in the present study, we observed differences in antimicrobial susceptibility and distribution of ARGs among different lineages. These results may reflect the potential association between the core genes and ARGs. Especially in clade 1, these strains, isolated from different regions and sources, are enriched with multiple ARGs. Recently, the interaction between the core genes and accessory genes has been observed in the pathogen *Vibrio parahaemolyticus*, suggesting that it may contribute to the adaptation of the bacterium to different marine ecological environments (57). In addition, several studies have confirmed the existence of patterns of co-occurrence and avoidance between genes (58–60). Nevertheless, we lack evidence to confirm that the potential association between the core genome and resistance genes is mediated by direct gene-gene interactions rather than arising due to selection pressure from the external environment. For example, we observed that strains belonging to clade 11 were more susceptible to antibiotics and carried few resistance genes. Notably, these clade 11 strains were predominantly isolated from the respiratory tract. Therefore, the characteristics observed in these strains may be attributed to the specific ecological niche they inhabit.

In the present study, AST and WGS were used to characterize the AMR profiles and ARGs of *R. anatipestifer* isolates collected from China and other locations globally. Our results demonstrated the severe resistance status of *R. anatipestifer* and the extremely high carriage of resistance genes. Importantly, our results highlight the prevalence of *tet*(X) and beta-lactamase genes in *R. anatipestifer* and reveal the uneven distribution of resistance genes among lineages. Overall, this study provides comprehensive insights into the AMR profile of the *R. anatipestifer* population in mainland China.

## Data Availability

All genomic data generated in this study are available under the NCBI BioProject accession number PRJNA705533. The accession numbers of the genomic assembly used in this study can be found in Table S1.

## SUPPLEMENTAL MATERIAL

Supplemental figures spectrum.03132-23-s0001.pdf Fig. S1 and S2.

Supplemental tables spectrum.03132-23-s0002.xlsx Tables S1 to S4.

ASM does not own the copyrights to Supplemental Material that may be linked to, or accessed through, an article. The authors have granted ASM a non-exclusive, world-wide license to publish the Supplemental Material files. Please contact the corresponding author directly for reuse.
