# Supplementary material for: Genome-based assessment of antimicrobial resistance reveals the lineage specificity of resistance and resistance gene profiles in Riemerella anatipestifer from China
Source: Microbiol Spectr. 2024 Jan 3;12(2):e03132-23. doi: 10.1128/spectrum.03132-23 (PMC10846147; doi:10.1128/spectrum.03132-23)
Supplement: Supplemental figures — Fig. S1 and S2. [file spectrum.03132-23-s0001.pdf]

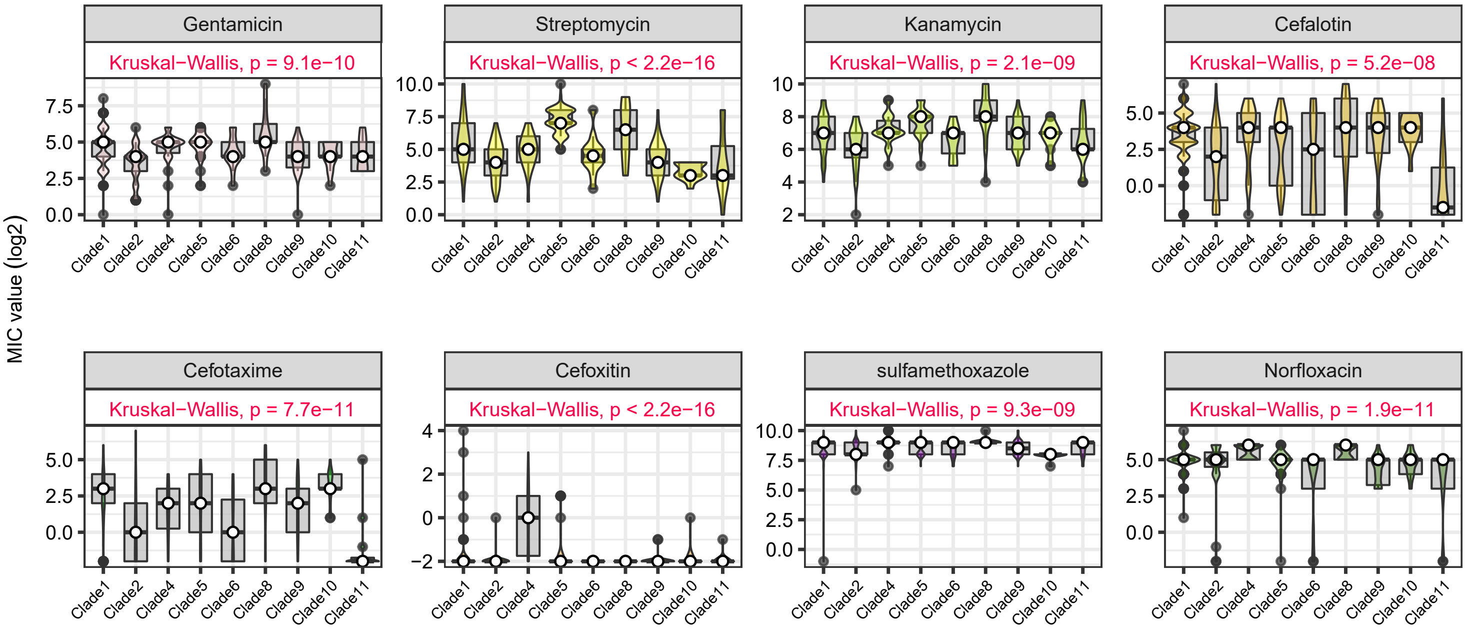

Figure S1. Comparison of MIC distributions among different lineages.The Kruskal-Wallis rank-sum test was applied for multiple group comparisons Eleven clades were identified according to the hierarchical Bayesian analysis by FastBaps (https://github.com/gtonkinhill/fastbaps) based on core genome alignment. We only analysed clades with more than 10 strains.

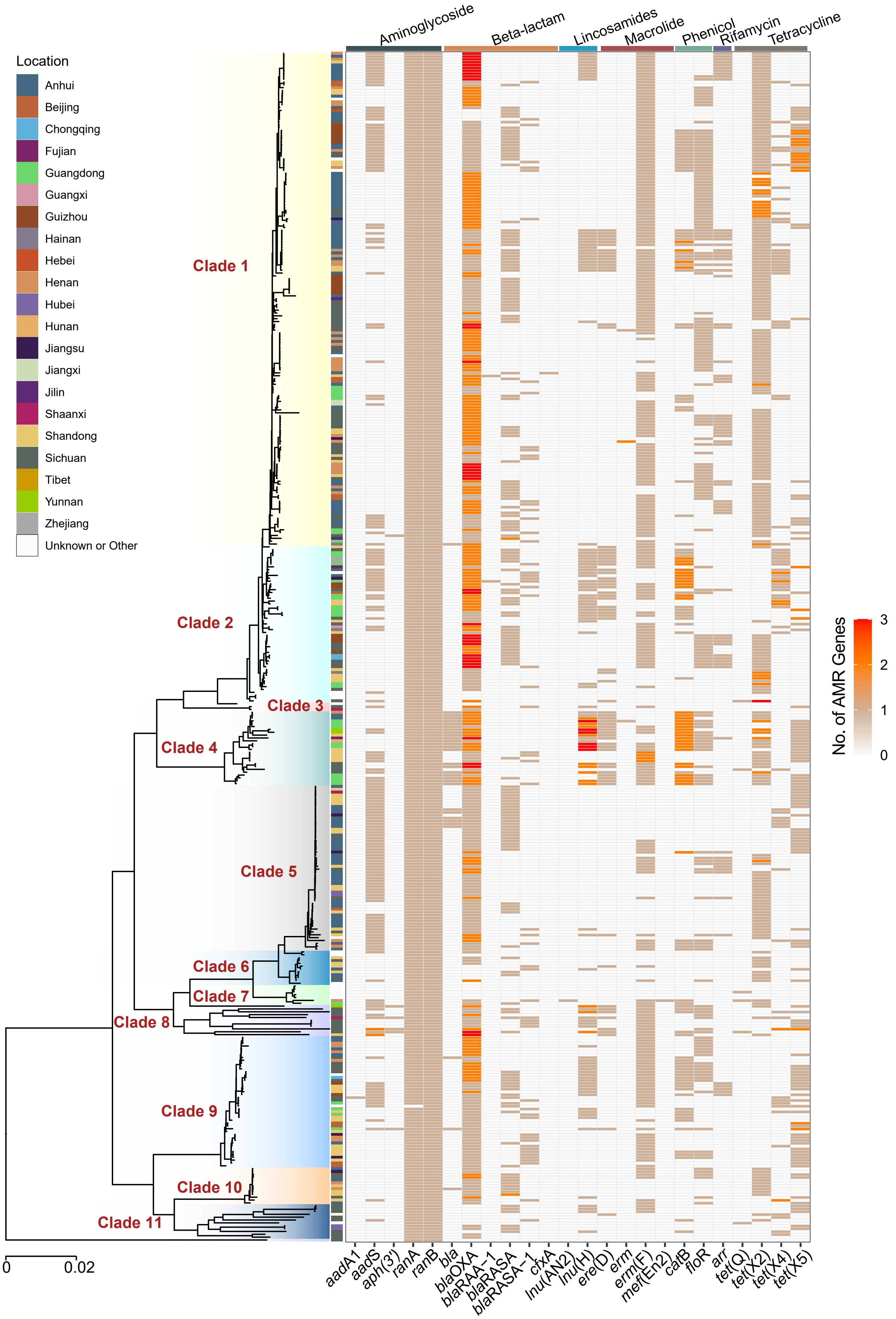

Figure S2. Distribution of antimicrobial resistance phenotypes, antimicrobial resistance genes. A core-genome-based neighbor joining(NJ) tree was conducted using PopPUNK. Eleven clades were identified according to the hierarchical Bayesian analysis by FastBaps (https://github.com/gtonkinhill/fastbaps) based on core genome alignment.
